# Performance of a convolutional neural network derived from an ECG database in recognizing myocardial infarction

**DOI:** 10.1038/s41598-020-65105-x

**Published:** 2020-05-21

**Authors:** Hisaki Makimoto, Moritz Höckmann, Tina Lin, David Glöckner, Shqipe Gerguri, Lukas Clasen, Jan Schmidt, Athena Assadi-Schmidt, Alexandru Bejinariu, Patrick Müller, Stephan Angendohr, Mehran Babady, Christoph Brinkmeyer, Asuka Makimoto, Malte Kelm

**Affiliations:** 1 Division of Cardiology, Pulmonology and Vascular Medicine, Medical Faculty, Heinrich-Heine-University Düsseldorf, Duesseldorf, Germany; 2 Cardiovascular Research Institute Düsseldorf (CARID), Medical Faculty, Heinrich-Heine-University Düsseldorf, Duesseldorf, Germany; 3 GenesisCare, Victoria, Australia

**Keywords:** Information technology, Myocardial infarction

## Abstract

Artificial intelligence (AI) is developing rapidly in the medical technology field, particularly in image analysis. ECG-diagnosis is an image analysis in the sense that cardiologists assess the waveforms presented in a 2-dimensional image. We hypothesized that an AI using a convolutional neural network (CNN) may also recognize ECG images and patterns accurately. We used the PTB ECG database consisting of 289 ECGs including 148 myocardial infarction (MI) cases to develop a CNN to recognize MI in ECG. Our CNN model, equipped with 6-layer architecture, was trained with training-set ECGs. After that, our CNN and 10 physicians are tested with test-set ECGs and compared their MI recognition capability in metrics F1 (harmonic mean of precision and recall) and accuracy. The F1 and accuracy by our CNN were significantly higher (83 ± 4%, 81 ± 4%) as compared to physicians (70 ± 7%, 67 ± 7%, P < 0.0001, respectively). Furthermore, elimination of Goldberger-leads or ECG image compression up to quarter resolution did not significantly decrease the recognition capability. Deep learning with a simple CNN for image analysis may achieve a comparable capability to physicians in recognizing MI on ECG. Further investigation is warranted for the use of AI in ECG image assessment.

## Introduction

**Figure 1:**
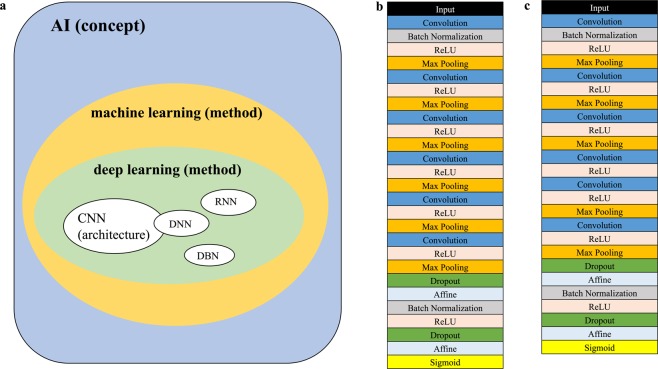
Concept and structures of Neural Networks. (**a**) A concept of artificial intelligence (AI). AI encloses machine learning and deep learning as methodologies. Deep learning includes neural networks as architectures. (**b**) The convolutional neural network (CNN) structure which were used to analyze the optimal number of ECG leads is shown. Six convolutional layers with relu activation and max-pooling layer were followed by a linear output layer into a sigmoid. (**c**) The CNN structure which were used to evaluate the effect of ECG image-quality reduction is shown. The fourth max-pooling layer was omitted as compared to Fig. 1b due to the pixel number of input ECGs.

Artificial intelligence (AI) is developing at a remarkable rate in parallel to computational advancement. Although a solid definition for an AI has not yet been established, AI is a concept which simulates human intelligence processes. This encompasses machine learning, deep learning as methods, and neural networks as architectures (Fig. 1a).

In the medical field, its diagnostic and possible therapeutic capabilities have also been rapidly developed and incorporated^1^. In the field of cardiology, the usefulness of AI for echocardiography and also for electrocardiography (ECG) has recently been reported using big data^2,3^.

On the other hand, practical use of these technologies is still on the way, due to local computational data and human capability resources.

In this study, we have assessed the usefulness of an AI, which is comprised of a simple neural network architecture, and trained by the ECGs as “images”, not by electric signals, based on a relatively small ECG database, for diagnosing myocardial infarction (MI). We hypothesized that an AI with a simple neural network architecture using even a small database as image analysis will be even a small database for image analysis may be non-inferior to human physicians and cardiologists in recognizing MI. We also evaluated the influence of ECG-lead reduction and image compression on the recognition capability of the AI. This research will assess the feasibility of the practical introduction of these AI systems in the setting of moderate computational facilities and resources.

## Methods

### Structure of the convolutional neural network (CNN)

The structures of our convolutional neural networks (CNN) are shown in Figs. 1b and 1c. We adopted a 6-layered CNN in the present study (Fig. 1b) based on our previous experiments (Suppl. Table 1). For the trial with ECGs with various image qualities, we developed a variant of the normal 6-layered CNN reducing one max-pooling-layer in order to make the program suitable for the input data volume of ECGs (Fig. 1c).

### Electrocardiography sampling

**Figure 2:**
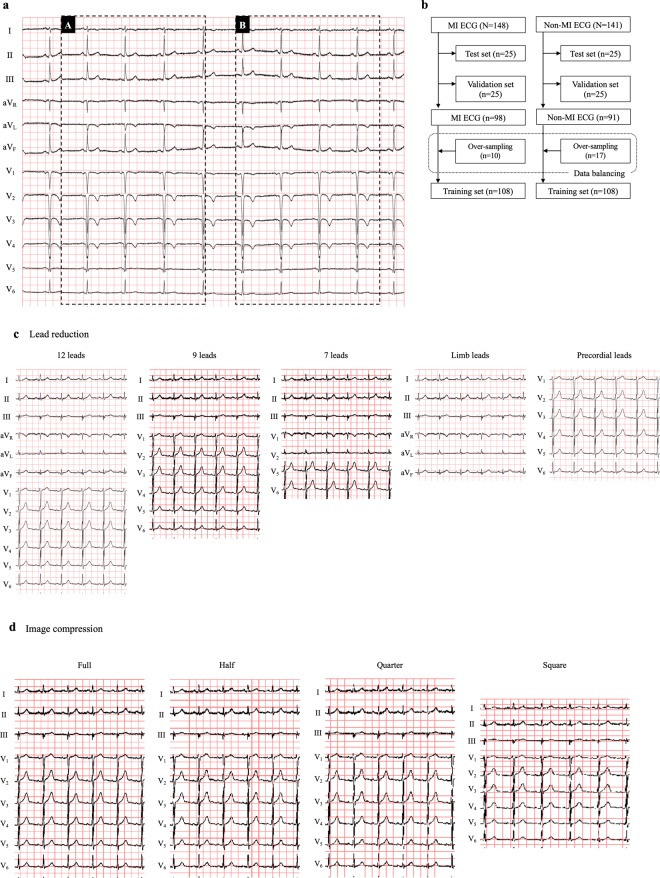
ECG data and datasets preparation. (**a**) Target ECG regions for the present study are shown. The region A (744*368) was extracted for our deep learning processes. The region B (744*368) was used for over-sampling in order to resolve the data imbalance (see text in detail). (**b**) Dataset preparation is shown in this flowchart. As test sets and validation sets, 25 ECGs were randomly selected respectively. For data balancing, 10 or 17 ECGs were randomly selected in each group (over-sampling, see Fig. 2a), and added to construct the training sets. (**c**) ECGs with lead reduction are shown. 9-lead-ECG consisted of leads I, II, III, and V_1–6_. 7-lead-ECG consisted of leads I, II, III, V_1–2_, and V_5–6_. (**d**) Based on the 9-lead-ECG, we compressed its image quality in 4 different ratios; full quality (744*368), half quality (398*260), quarter quality (282*184) and square-shape (224*224).

We used the PTB diagnostic ECG database (https://www.physionet.org/content/ptbdb/1.0.0/) which contains 549 records of 12-lead-ECGs from 290 patients in PNG format (1183*983)^4,5^. This database has already been published online by a third party unrelated to the study, therefore there should be no concerns regarding the ethical disclosure of the information. We downloaded all ECGs within this database and pre-processed them for our study (PHYSIOBANK ATM: https://archive.physionet.org/cgi-bin/atm/ATM). We extracted 289 ECGs choosing one ECG per patient except for 1 patient who did not have an ECG on record. The extracted ECGs consist of 148 ECGs with the diagnosis of acute myocardial infarction (MI) with its infarct territory localization information and 141 ECGs without MI (non-MI) (Table 1). To prepare the dataset for each trial, a certain section of image in PNG format (744*368) was clipped out from each ECG (offsets: top 36, left 202, bottom 780, right 570) as our original dataset (Fig. 2a). From this original dataset we have randomly chosen test-sets (n = 25) and then validation-sets (n = 25). After that, the sample number was balanced between MI and non-MI by over-sampling to gain training-sets (n = 108, each) (Fig. 2b**)**. For over-sampling we clipped out the ECG image in the same size (PNG format, 744*368, offsets: top 36, left 664, bottom 780, right 1032) as the other region, where it had not been used for our original dataset (Fig. 2a). To assess the reproducibility, we prepared 10 different training/validation/test ECG sets as well from our original dataset by random- and over-sampling^6^.

**Table 1:** Diagnosis in the PTB Database.

Diagnosis		N = 289
Myocardial infarction (MI)		148
Anterior MI		72
Inferior MI		74
Posterior MI		15
Septal MI		30
Lateral MI		55
Non-MI	Healthy control	52
	Cardiomyopathy	15
	Bundle branch block	15
	Dysarrhythmia	14
	Hypertrophy	7
	Valvular heart disease	6
	Myocarditis	4
	Heart failure	3
	Angina pectoris	3
	Others	22

### Lead-set and image-quality comparisons

We built the variants of training/validation/test ECG sets from our original ECG datasets that differed by the number of the leads (Fig. 2c) or in its image-quality (Fig. 2d). For our lead-sets construction, we prepared 5 variations all in full image quality; original (12-leads), 9-, 7-, limb-, and precordial-leads. 9-lead-ECG consisted of leads I, II, III, and V_1–6_. 7-lead-ECG consisted of leads I, II, III, V_1–2_, and V_5–6_.

For our image-quality variations we adopted the 9-lead-ECG and compressed its image quality in 4 different ratios; full quality (744*368), half quality (398*260), quarter quality (282*184) and square-shape (224*224).

### Training a convolutional neural network to recognize an ECG with MI

We developed a 6-layered convolutional neural network as our CNN model for the present study. We used a deep learning architecture which is a form of machine learning devised to mimic the visual system^7^. For each training/validation/test ECG set, this CNN model was trained from a training-set of 216 ECG-images (MI: non-MI = 108:108) with the result of “Yes (MI)” or “No (non-MI)” paralleled with validation using a validation-set (MI: non-MI = 25:25). This validation-set usage was intended to avoid over-fitting during the network training. During only the network training, we incorporated data augmentation (random rotation [−4 to 4 degrees], width-shift [±5%], shear transformation [shear angle in counter-clockwise direction: up to 0.05 degrees]) to increase the learning efficacy. The areas outside the boundaries of the input are filled according to the ‘nearest’ mode. As methods of data augmentation, flip, zoom and brightness variations were not adopted. After the training, we tested the generalized capability of the CNN model with the test-sets of 50 ECGs (MI: non-MI = 25:25). The output of this model is Yes (MI) or No (non-MI) to the unknown ECGs, i.e. ECG-images not used during the training (Fig. 2). We repeated this training and test process 10 times for 10 different training/validation/test ECG sets to confirm its reproducibility. The training of the CNN was independently conducted for each training/validation/test ECG set and each variant of training/validation/test ECG set (lead reduction, image compression) based on the validation loss to adopt a model with the least validation loss.

### Assessments of accuracy

For the metrics to assess the capability of the CNN or physicians to recognize ECGs, we adopted the F1 measure (harmonic mean of sensitivity and positive predictive value) and the rate of accuracy as a percentage. The area under the curve (AUC) of receiver-operator-characteristic (ROC) curve was also assessed. These metrics were computed for each training/validation/test ECG set (n = 10).

Ten physicians were tested with 2 test-sets of 12-lead-ECG image (100 ECGs) out of 10 test-sets which were also used during the tests for CNN. Five of them (including 3 consultants) are board-certified, 3 are senior residents and the other 2 physicians are junior residents with more than 1 year of training in the cardiology. All 10 physicians have been engaged in the emergency medical care including management of acute coronary syndrome. Each test-set was ensured to be assigned to one board-certified cardiologist and one non-board-certified physician. For each test-set, the pairs of one board-certified cardiologist and one non-board-certified physician were also randomly assigned. They judged whether the patient of each ECG had experienced a MI or not. The majority opinion of physicians for each record were aggregated and was taken as reference physicians’ consensus. If the opinions were evenly divided, the opinion of board-certified cardiologists was counted with 2x weight.

Additionally, we randomly chose 72 ECGs and all ten cardiologists judged whether these 72 patients had experienced a MI or not. This assessment was used to find out for which ECG all cardiologists made correct or false judgements.

Data on infarction sites (anterior, inferior, posterior, septal and lateral) were also extracted from the PTB diagnostic ECG database (Table 1). The detection accuracy according to these infarction sites was also assessed.

### Visualization of the features identified by deep learning

We generated activation maps of the final convolutional layer using Grad-CAMs which illustrated the relative positive activation of a convolutional layer with respect to the network output^8^.

### Statistical analysis

Continuous data were shown as mean ± SD for normally distributed data. In cases of non-normal distributed data, they were shown as median values (lower-upper quartile). Categorical data were shown as numbers and percentages. The chi-square test, Kruskal-Wallis test, Student t test, Fisher’s exact test, or 1-way analysis of variance was performed when appropriate. Post-hoc analysis using Turkey’s HSD Test was conducted if a multivariate test revealed statistical significance. For global test statistics, we used a significance level of 5% (two tailed). Analyses were performed using python 3.6.0 or JMP (SAS, Version 11).

## Results

### Recognition capability of the ECGs with different lead-settings

**Figure 3:**
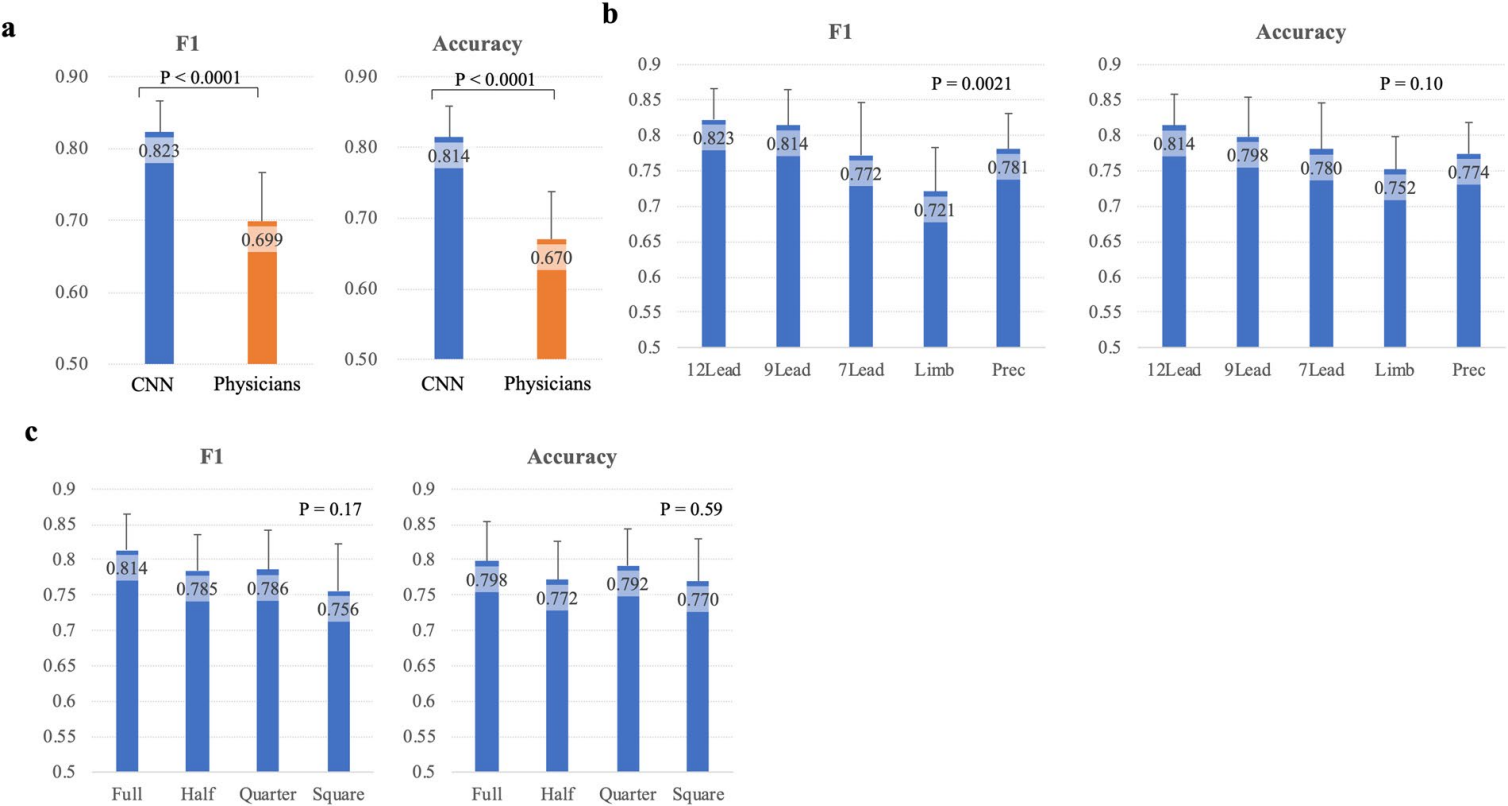
Comparisons of recognition capabilities. Recognition capabilities are shown in 2 representative metrics (F1 measure = harmonic mean of sensitivity and positive predictive value; accuracy = degree to which the result of the CNN prediction conforms to the correct classification). (**a**) The comparison between the convolutional neural network (CNN) and human cardiologists is shown. The recognition capability of the 6-layer CNN was significantly higher than that of human cardiologists (F1 measure 0.788 ± 0.056 vs 0.699 ± 0.068, P < 0.0001; accuracy 0.788 ± 0.052 vs 0.67 ± 0.067, P < 0.0001). (**b**) The impact of ECG lead reduction. The 6-layer CNN with limb-lead ECGs showed significantly lower F1 measure as compared to that with 12-lead and 9-lead ECGs (0.721 ± 0.062 vs 0.823 ± 0.043, P = 0.0022; 0.721 ± 0.062 vs 0.814 ± 0.051, P = 0.0058, respectively). The ECG lead reduction did not significantly affect accuracy. (**c**) The impact of ECG image quality. The recognition capability of the 6-layer CNN showed no significant differences with reduced ECG image quality.

The recognition capabilities of our CNN and physicians to the full-quality standard 12-lead-ECG are shown in Fig. 3a. The CNN achieved significantly higher F1 and accuracy as compared to physicians (83 ± 4% vs. 70 ± 7%, p < 0.0001; 81 ± 4% vs. 67 ± 7%, p < 0.0001). The sensitivity (86 ± 7% vs 67 ± 10%, p < 0.0001), the specificity (76 ± 7% vs. 67 ± 10%, p = 0.018), and the negative predictive value (85 ± 7% vs. 60 ± 10%, p < 0.0001), were significantly higher with our CNN than physicians (**Suppl**. Figure 1). The positive predictive value of our CNN tended to be higher as compared to that of physicians (79 ± 5% vs. 74 ± 6%, p = 0.053). Even if we compared these metrics of the CNN with those of board-certified cardiologists, the capabilities of the CNN were higher or not inferior as compared to those of board-certified cardiologists (**Suppl**. Figure 2). The F1 and accuracy of the consensus of physicians were not superior to the average of single physicians (0.67 and 0.69, respectively; Suppl. Table 2).

In regards to the MI detection according to the infarction site, the accuracy in the MI cases in the test-ECG sets was assessed (Suppl. Table 3). There were no significant differences in the detection accuracy depending on the infarction site (P = 0.27).

The recognition capability of our CNN with reduced ECG leads are shown in Fig. 3b and Table 2. The F1 measure of the CNN trained with limb-lead-ECGs (72 ± 6%) was significantly lower as compared to that with 12-lead-ECGs (82 ± 4%, p = 0.0022) and 9-lead-ECGs (81 ± 5%, p = 0.0058). There was however no significant difference in accuracy between the number of ECG leads used (p = 0.10). All CNN read ECGs except when only the limb-lead-ECG were used showed significantly higher F1 and accuracy than human physicians (p < 0.05). There were no significant differences in test loss values and AUC between the CNNs with different numbers of ECG leads (Table 2). There were also no significant differences in the accuracy and AUC of training- and validation-sets at the end of the training.

**Table 2:** Metrics of recognition capability according to number of leads.

	12 lead	9 lead	7 lead	Limb lead	Prec. lead	P value
Sensitivity	0.86 ± 0.07	0.88 ± 0.07	0.75 ± 0.10	0.65 ± 0.09	0.81 ± 0.09	<0.0001
Specificity	0.76 ± 0.07	0.71 ± 0.10	0.81 ± 0.10	0.86 ± 0.07	0.74 ± 0.09	0.0047
PPV	0.79 ± 0.05	0.76 ± 0.07	0.80 ± 0.08	0.82 ± 0.06	0.76 ± 0.06	0.13
NPV	0.85 ± 0.07	0.87 ± 0.08	0.77 ± 0.07	0.71 ± 0.05	0.80 ± 0.06	<0.0001
F1	0.82 ± 0.04	0.81 ± 0.05	0.77 ± 0.07	0.72 ± 0.06	0.78 ± 0.05	0.0021
Accuracy	0.81 ± 0.04	0.80 ± 0.06	0.78 ± 0.07	0.75 ± 0.05	0.77 ± 0.04	0.10
Loss	1.56 ± 0.31	1.50 ± 0.19	1.41 ± 0.23	1.44 ± 0.18	1.41 ± 0.20	0.55
AUC	0.88 ± 0.05	0.88 ± 0.04	0.86 ± 0.05	0.85 ± 0.05	0.87 ± 0.04	0.45
Accuracy (training)	0.89 ± 0.06	0.91 ± 0.05	0.88 ± 0.06	0.88 ± 0.04	0.88 ± 0.03	0.39
AUC (training)	0.97 ± 0.03	0.98 ± 0.02	0.96 ± 0.03	0.96 ± 0.03	0.97 ± 0.01	0.29
Accuracy (validation)	0.85 ± 0.06	0.85 ± 0.06	0.82 ± 0.05	0.82 ± 0.03	0.82 ± 0.03	0.40
AUC (validation)	0.93 ± 0.05	0.93 ± 0.05	0.91 ± 0.06	0.92 ± 0.05	0.92 ± 0.05	0.89

### Recognition capability with compressed image quality

The recognition capability of our CNN to read ECGs with various image qualities are shown in Fig. 3c. The reduction of image quality up to a quarter or even a change of the aspect ratio of the ECG did not affect F1 or accuracy.

As shown in Table 3, the sensitivity of CNN to read ECGs in full or half image quality was significantly higher than that with square shaped ECGs (88 ± 7%, 84 ± 10% vs. 72 ± 10%, p = 0.0022, 0.042, respectively). On the other hand, the specificity of CNN to read square shaped ECGs tended to be higher than that with ECGs in half image quality (82 ± 9% vs. 71 ± 12%, p = 0.091). The test loss of CNN was significantly higher with half image quality ECGs as compared to the other 3 models. There was no significant difference in the test AUC between the models with the different image qualities. The performance in the validation-sets, particularly the AUC, was significantly lower with square-shape ECGs.

**Table 3:** Metrics of recognition capability according to image quality.

	Full	Half	Quarter	Square	P value
Sensitivity	0.88 ± 0.07	0.84 ± 0.10	0.77 ± 0.10	0.72 ± 0.10	0.0021
Specificity	0.71 ± 0.10	0.71 ± 0.12	0.82 ± 0.10	0.82 ± 0.09	0.022
PPV	0.76 ± 0.07	0.75 ± 0.08	0.82 ± 0.09	0.81 ± 0.09	0.19
NPV	0.87 ± 0.08	0.82 ± 0.09	0.79 ± 0.07	0.75 ± 0.07	0.016
F1	0.81 ± 0.05	0.78 ± 0.05	0.79 ± 0.06	0.76 ± 0.07	0.17
Accuracy	0.80 ± 0.06	0.77 ± 0.05	0.79 ± 0.05	0.77 ± 0.06	0.59
Loss	1.50 ± 0.19	1.77 ± 0.28	1.48 ± 0.18	1.46 ± 0.22	0.0082
AUC	0.88 ± 0.04	0.87 ± 0.05	0.87 ± 0.05	0.85 ± 0.05	0.48
Accuracy (training)	0.91 ± 0.04	0.92 ± 0.04	0.86 ± 0.05	0.83 ± 0.06	0.0003
AUC (training)	0.98 ± 0.02	0.99 ± 0.02	0.95 ± 0.04	0.93 ± 0.05	0.0004
Accuracy (validation)	0.85 ± 0.06	0.81 ± 0.07	0.80 ± 0.07	0.79 ± 0.07	0.17
AUC (validation)	0.93 ± 0.05	0.92 ± 0.04	0.87 ± 0.07	0.85 ± 0.08	0.016

### What do our CNNs see?

**Figure 4:**
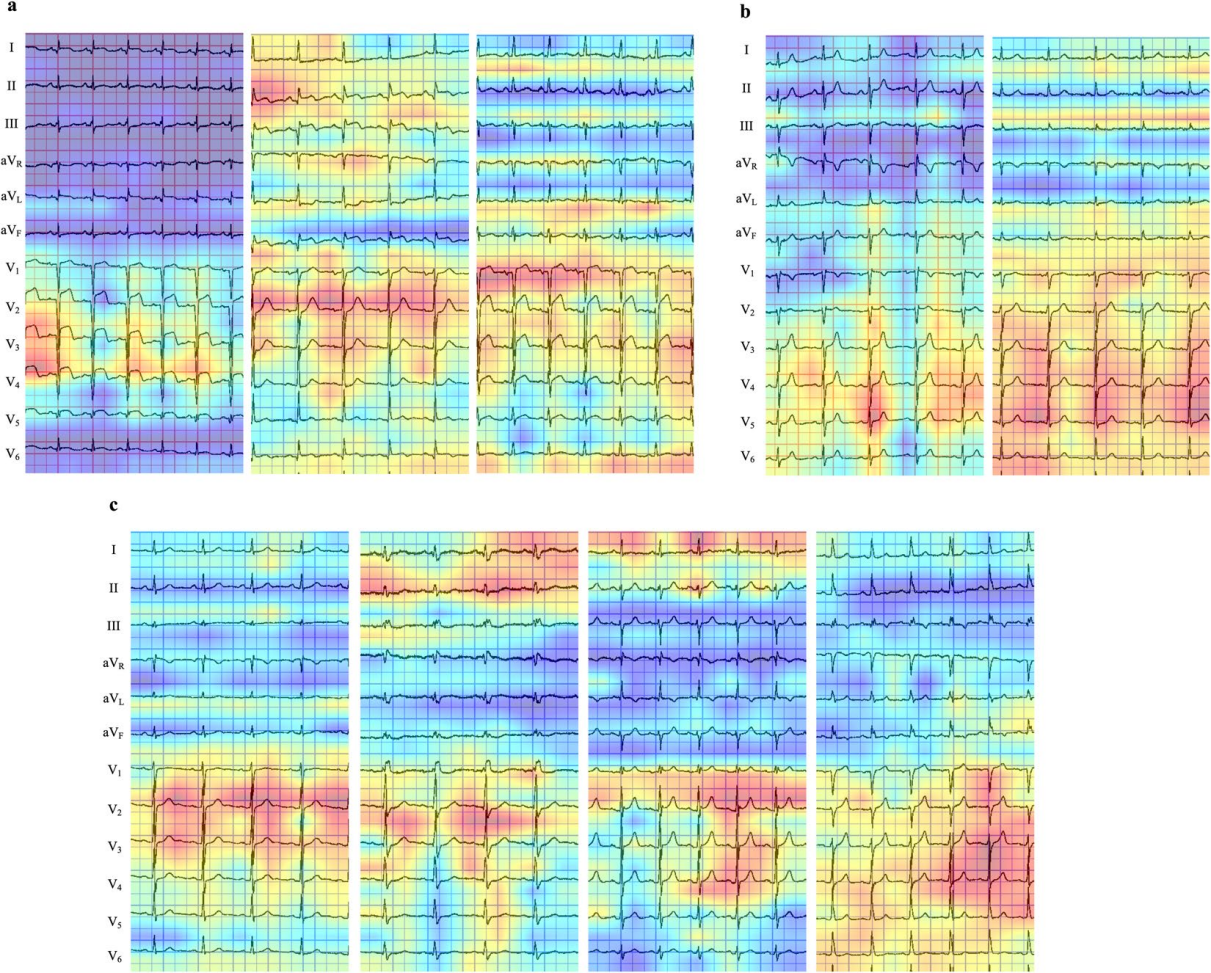
Heatmaps of last convolution layer activations. The heatmaps of the last convolution layer activity show from where the convolutional neural network (CNN) distinguished MI or non-MI in ECGs. The CNN focused on the red-colored zones. (**a**) Three cases with MI, in which both the CNN and cardiologists achieved accurate classification. The CNN focused on the elevated ST-T segments. (**b)** Two cases without MI, in which both the CNN and cardiologists made the correct classification. The CNN distinguished these ECGs as non-MI based mainly on the QRS complex in the precordial leads. (**c**) Four cases with MI, in which only the CNN achieved accurate classification. The CNN seemed to focus on the ST-T segments mainly in the precordial leads and partially in the limb leads. These MI cases did not show typical ST-T segment change (elevation/depression) or large Q wave, which made recognition of MI only based on ECGs challenging.

We used the Grad-CAM to visualize the spots in ECGs on which the last convolutional layer of our CNNs focused on during its recognition (Fig. 4). In an ECG of MI cases (Fig. 4a), the CNN focused strongly on the ST-T elevation. In non-MI cases, the CNN focused broadly on the area from QRS complexes to ST-T segment (Fig. 4b).

In the additional experiment using 72 ECGs, all ten physicians could not make a diagnosis correctly in 8 cases (11.1%). These 8 ECGs were derived from either acute coronary syndromes (non-ST-segment-elevation MI, n = 6), or of old inferior MI (n = 2). One case with old inferior MI had complete right-bundle-branch block. The CNN models achieved average of 87.5 ± 11.8% accuracy for these 8 ECG records. In these cases, the CNN also focused strongly on the ST-T segments and QRS complexes (Fig. 4c).

## Discussion

The major findings of the present study were that 1) the artificial intelligence utilizing a 6-layered convolutional neural network achieved a higher capability in recognition of myocardial infarction ECG-images as compared to physicians. Of note, the sensitivity and the negative predictive value by our CNN were significantly higher as compared to those by physicians (**Suppl**. Figure 1), which suggest the clinical use to reduce misdiagnosis. 2) as to the number of ECG leads, its reduction did not significantly affect the recognition capability of the CNN with the choice of ECG leads in our experiments, 3) the image quality, even when compressed to a quarter quality, did not significantly affect the recognition capability of the CNN, which indicate the transferability of this architecture, and 4) the CNN focused on the ST-T elevation for the recognition of MI in ECGs as cardiologists do.

To the best of our knowledge, this is the first report directly comparing the capability of deep learning trained from a small dataset of ECG and human physicians. An automated, convenient and efficient artificial intelligence to read and diagnose ECGs should be imperative for emergency medicine, and our primary intention is to assess the feasibility of CNN architectures which can be achieved by commercially available and low-cost computational capabilities.

A previous study reported that deep learning achieved high recognition capability with the same PTB ECG database^9^. In their study, a 6-layer-CNN was incorporated using raw digital ECG data. The achieved sensitivity and specificity were about 0.90, higher as compared to our CNN with image analysis (Table 2). In order to improve these metrics for ECG as image analysis, further research is imperative. The performance level of human cardiologists in their report was based on the previous human analyzed data, not using ECGs from the PTB ECG database. In our study we incorporated 10 physicians, who were all currently clinically active and five of them are board-certified as cardiology specialists. Therefore, our results underscore the high performance of AI. Furthermore, we utilized the ECG as one image (PNG format) of multiple leads, not as raw digital data. This is exactly how cardiologists see and recognize ECG patterns in clinical practice.

Hannun *et al*. have reported that the AI utilizing CNN were at least as efficient as cardiologists in detecting arrhythmias^3^. They used the raw digital ECG data as input. In the present study we compared the efficiency of an “image recognition” task between human internists/cardiologists and AI. Recently Jun *et al*. successfully demonstrated the efficient arrhythmia classification by CNN with the input ECG as gray-scale images^10^. They used the ECG image of each single beat from one ECG strip, i.e. single lead for the purpose of detecting various arrhythmias using Holter-monitoring-ECG data. Our present AI makes a diagnosis based on the whole 12-lead-ECG and not only on one single beat in order to find previous MIs. Although the tasks of the AI were different, these results suggest the feasibility of CNN and their ECG image analysis.

Among the models with different layer construction (5, 6, and 7-layered), we have seen no significant difference in efficiency of deep learning but the necessary parameters were at the least in the 6-layered CNN model (Suppl. Table 1). We therefore adopted the 6-layered CNN model for the deep learning. Our CNN recognized MI ECGs more correctly than cardiologists, even trained with a small database (<300 ECGs). Our data demonstrated that using an AI with CNN to recognize pathological ECGs can be produced even without the need for big data and it can work at least as accurately as human cardiologists. The graphic processing unit (GPU) utilized for the present study was commercially available with 8 GB capacity. This allows easy access to this technology.

Among the ECGs with different lead-settings, only the configuration with only limb-lead ECGs resulted in lower recognition capability of the CNN compared to other lead-settings. In our results, the reduction of the recognition capability was not obvious except for limb-lead ECGs. However, the proportional reduction of the recognition capability according to the number of leads can be reasonable because the ECG abnormalities under MI appear in specific leads depending on the infarction sites. Even when our CNN trained with only limb-lead ECGs during this study, it still demonstrated a comparable recognition capability as cardiologists to the 12-lead-ECGs. That could suggest the possibilities to apply this technology to situations with limited medical resources or to the use of wearable devices.

We adopted ECGs in PNG format as input data, which is a common format to save images. This is practical in clinical situations, because ECGs are usually shown, diagnosed and preserved in 2-dimensional image format at present. There were no significant differences in the recognition capabilities of our CNN between the image qualities of input ECGs and even ECGs with lowered qualities resulted in a comparable recognition capability as cardiologists. That could suggest the possible uses of our CNN, for example to send the photographed ECGs to an emergency center equipped with the CNN for a medical diagnosis or even an application in mobile devices such as smartphones.

We visualized the CNN´s focus points on the ECGs during its recognition process with the heatmaps (Grad-CAM). It demonstrated that our CNN focused on the ST-T elevation in the ECG to judge MI, as did the cardiologists. In the MI-cases that only the CNN recognized correctly as MI, the CNN also focused on the ST-T segments. It was, however, difficult for cardiologists to judge MI from this focus area in the ECG in these cases. As shown in Fig. 4c, if there is no ST-T segment elevation/depression or Q waves, the recognition of MI, acute or old, becomes challenging. These heatmaps may provide us new criteria to read ECGs if we gather the heatmaps in ECGs in larger databases. On the other hand, in the present study we could not explain why the CNN focused on the ST-T segment of the certain beat (e.g. Figure 4a left panel and Fig. 4a middle panel). This should be further investigated using larger databases.

The CNN in the present study was trained with a small database and could make a diagnosis of MI on ECGs. Although it was already able to recognize ECGs as correctly as physicians/cardiologists, it could be more capable with more input data volume also as an image analysis, not based on the raw digital data. The ten models of CNN in this project showed consistently comparable to better recognition capabilities as compared to physicians/cardiologists, however the performance was slightly variable (**Suppl.** Figure 3) most probably due to the small data volume. Furthermore, with more input ECG data, not only for MI-cases but also for other diseases, we could expect to create an AI which uses ECGs to help diagnose a more extensive range cardiovascular diseases in the future.

We admit the computational limitation with our GPU capacity for deep learning. The feasibility and reliability should be confirmed in future research. Due to the exploratory feature of this study, sample size calculation had been not performed. We did not adopt multi-fold cross validation because of the imbalance of both arms and the image augmentation process. Instead, we generated 10 different training/validation/test ECG sets and averaged the results of the analysis. Although in the published database (PTB Diagnostic ECG Database) the assigned diagnosis of MI or non-MI were classified based on the subsequent clinical assessments including cardiac catheterization, further detailed clinical information was not available. Although Grad-CAM was adopted to visualize the focus of CNN in the present study, the visualizations are up-sampled based on the gradients of the last convolution layer activation. Therefore the resolution of focus should be carefully evaluated. Furthermore, it has been pointed out, that Grad-CAM can fail to properly localize objects in an image if the image contains multiple occurrences of the same classification. This drawback could be overcome with further developments of algorithms to visualize foci of neural networks^11^. Finally, this research was not intended to demonstrate inferiority of human physicians/cardiologists. We recognize that a diagnosis is not only based purely on ECG interpretation but also on clinical assessment, which is not feasible for AI systems at least at this time. However, this technology could be used as a first line triage and assessment tool to aid in the final clinical diagnosis.

## Conclusions

Deep learning with a simple convolutional neural network architecture derived from even a small ECG database may provide an advantage or at least non-inferior to cardiologists in recognizing myocardial infarction. The removal of the Goldberger-leads or image compression up to quarter resolution does not significantly affect the recognition capability. Further investigation is warranted to establish whether the use of AI to assess ECGs as a triage or risk stratification tool is clinically feasible.

## Data availability

The utilized data (PTB ECG Databank) are all available in the Physionet (has been freely published). The authors have no restrictions for disclose the utilized ECGs or information on 10 groups of randomized ECG sets.

## Supplementary information

Supplementary Figure 1 .

Supplementary Figure 2.

Supplementary Figure 3.

Supplementary information.
