# Supplementary figures and images for: Performance of a convolutional neural network derived from an ECG database in recognizing myocardial infarction
Source: Sci Rep. 2020 May 21;10:8445. doi: 10.1038/s41598-020-65105-x (PMC7242480; doi:10.1038/s41598-020-65105-x)

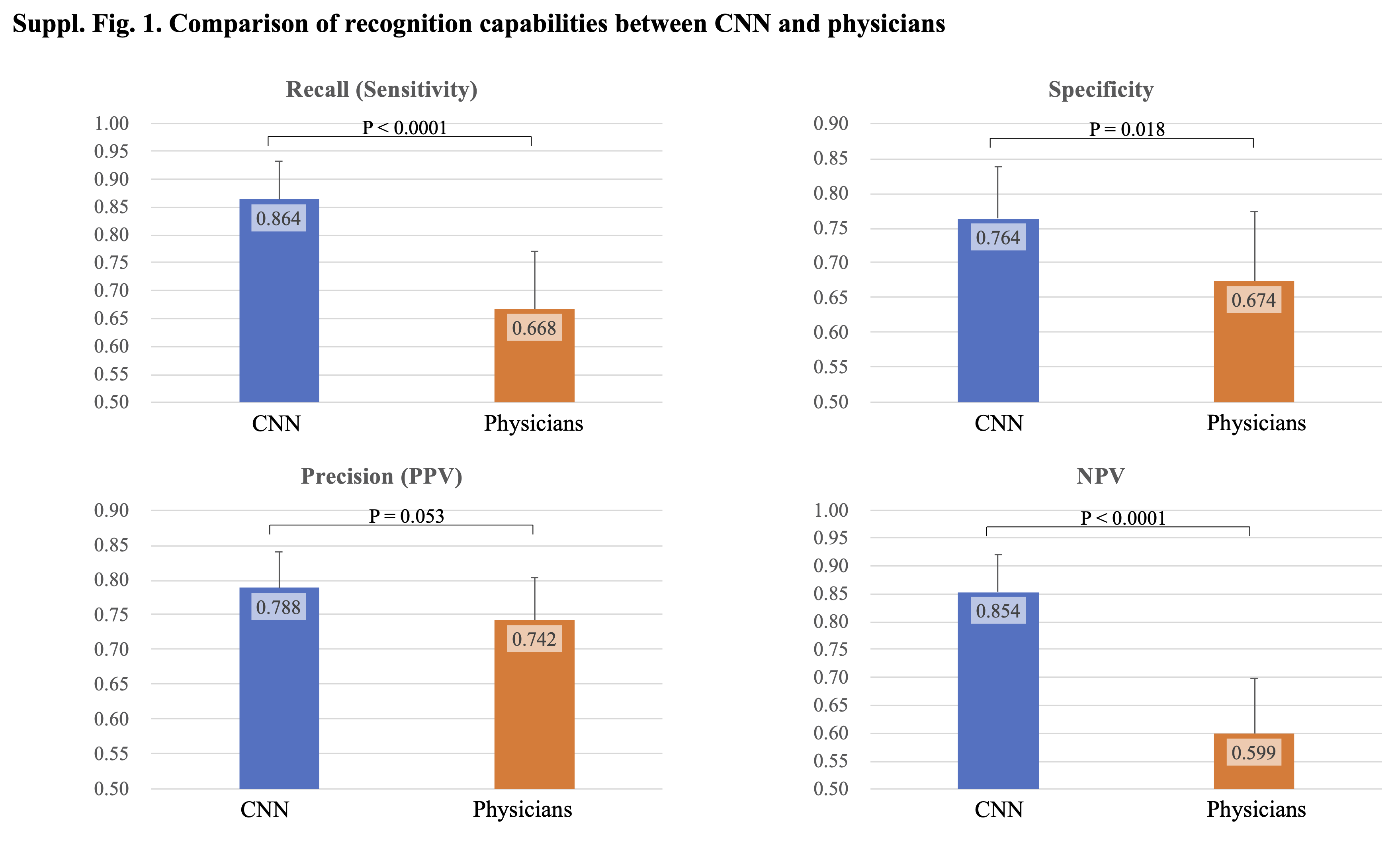

Supplement: Supplementary file 1 — Supplementary Figure 1 . [file 41598_2020_65105_MOESM1_ESM.tif]

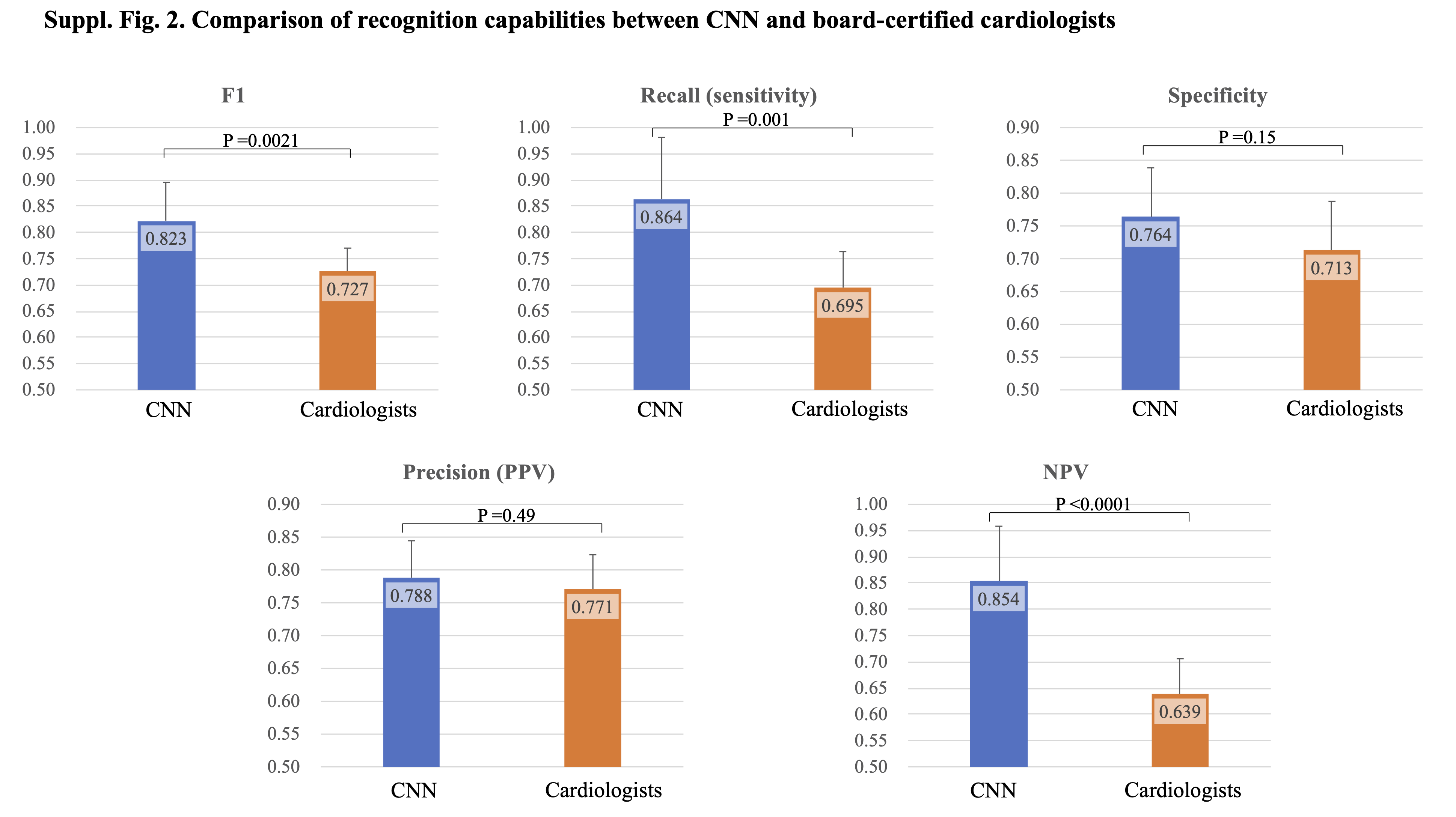

Supplement: Supplementary file 2 — Supplementary Figure 2. [file 41598_2020_65105_MOESM2_ESM.tif]

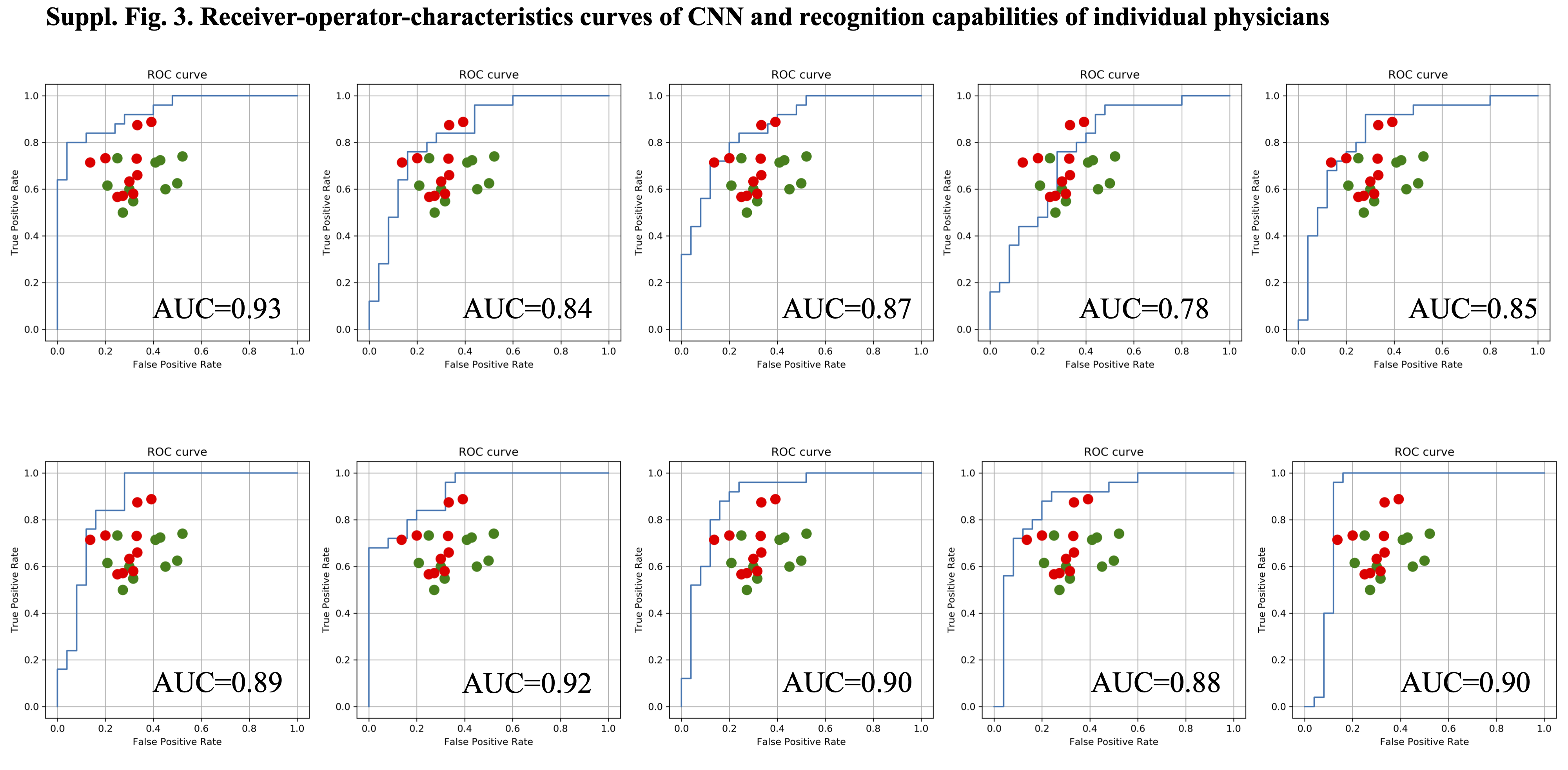

Supplement: Supplementary file 3 — Supplementary Figure 3. [file 41598_2020_65105_MOESM3_ESM.tif]
