# Supplementary material for: Performance of a convolutional neural network derived from an ECG database in recognizing myocardial infarction
Source: Sci Rep. 2020 May 21;10:8445. doi: 10.1038/s41598-020-65105-x (PMC7242480; doi:10.1038/s41598-020-65105-x)
Supplement: Supplementary file 4 — Supplementary information. [file 41598_2020_65105_MOESM4_ESM.docx]

**Supplementary Materials**

**Performance of a convolutional neural network derived from an ECG database in recognizing myocardial infarction**

Hisaki Makimoto*, MD, PhD 1,2; Moritz Höckmann 1; Tina Lin, MBBS BMedSci FRACP 3; David Glöckner, MD 1; Shqipe Gerguri, MD 1; Lukas Clasen, MD 1; Jan Schmidt, MD 1; Athena Assadi-Schmidt, MD 1; Alexandru Bejinariu, MD 1; Patrick Müller, MD 1; Stephan Angendohr, MD 1; Mehran Babady, MD 1; Christoph Brinkmeyer, MD 1; Asuka Makimoto 1; Malte Kelm, MD 1,2

1. Division of Cardiology, Pulmonology and Vascular Medicine, Medical Faculty, Heinrich-Heine-University Düsseldorf, Duesseldorf, Germany

2. Cardiovascular Research Institute Düsseldorf (CARID), Medical Faculty, Heinrich-Heine-University Düsseldorf, Germany

3. GenesisCare, Victoria, Australia

**Short title**: Efficient ECG recognition using AI

**Corresponding Author (*)**:

Hisaki Makimoto, M.D., Ph.D.

Division of Cardiology, Pulmonology and Vascular Medicine, Medical Faculty, Heinrich-Heine-University, Moorenstrasse 5, 40225 Düsseldorf, Germany

Phone: +49 211 81 18800, Fax: +49 211 81 19520

E-Mail: [h1sak1mak1m0t0@gmail.com](mailto:h1sak1mak1m0t0@gmail.com)

**Legends of Supplementary Figures**

**Suppl. Fig. 1**. Comparison of recognition capabilities between CNN and physicians

Sensitivity, specificity, positive predictive value (PPV) and negative predictive value (NPV) of the convolutional neural network (CNN) and physicians are shown. The recognition capabilities (sensitivity, specificity and NPV) of the CNN were significantly higher as compared to those of physicians (P<0.05). PPV of the CNN tended to be higher than that of physicians (P=0.053)

**Suppl. Fig. 2**. Comparison of recognition capabilities between CNN and board-certified cardiologists

F1, sensitivity, specificity, positive predictive value (PPV) and negative predictive value (NPV) of the convolutional neural network (CNN) and the board-certified cardiologists (n=5) are shown. The recognition capabilities (F1, sensitivity, and NPV) of the CNN were significantly higher as compared to those of cardiologists (P<0.05).

**Suppl. Fig. 3**. Receiver-operator-characteristics curves of CNN and recognition capabilities of individual physicians

The receiver-operator-characteristics curves of each model derived from 10 different training ECG sets of 12-lead-ECG are shown. Red-dots are the results of the board-certified cardiologists and green-dots are the results of the residents. Notably, the CNN models accomplished at least the comparable recognition capability as compared to physicians despite there were some variations in capability according to the different training ECG sets.

**Supplementary Tables**

**Suppl. Tab. 1**. Number of layers in CNN models

|  | Parameters | Accuracy | P value | F1 measure | P value | Loss | P value |
| --- | --- | --- | --- | --- | --- | --- | --- |
| CNN 5 layers | 17,711,745 | 0.818±0.054 | 0.24 | 0.825±0.053 | 0.28 | 1.82±0.33 | 0.15 |
| CNN 6 layers | 7,787,201 | 0.814±0.044 |  | 0.823±0.044 |  | 1.56±0.31 |  |
| CNN 7 layers | 7,824,129 | 0.784±0.046 |  | 0.793±0.051 |  | 1.59±0.30 |  |

**Suppl. Tab. 2.** Metrics of recognition capability of physicians

|  | **All Physicians** | **Non Board-Certified Physicians** | **Board-Certified Cardiologists** | **Majority Opinion of**  **All Physicians** |
| --- | --- | --- | --- | --- |
| Sensitivity | 0.67±0.10 | 0.64±0.08 | 0.69±0.12 | 0.67 |
| Specificity | 0.67±0.10 | 0.63±0.11 | 0.71±0.07 | 0.67 |
| PPV | 0.74±0.06 | 0.71±0.05 | 0.77±0.06 | 0.71 |
| NPV | 0.60±0.10 | 0.56±0.08 | 0.64±0.10 | 0.63 |
| F1 | 0.70±0.07 | 0.67±0.05 | 0.73±0.07 | 0.69 |
| Accuracy | 0.67±0.07 | 0.64±0.05 | 0.70±0.07 | 0.67 |

**Suppl. Tab. 3.** Accuracy in each ECG test-set according to infarction sites

|  | **Ant MI Acc**  **(correct / total)** | **Inf MI Acc**  **(correct / total)** | **Post MI Acc**  **(correct / total)** | **Septal MI Acc**  **(correct / total)** | **Lateral MI Acc**  **(correct / total)** |
| --- | --- | --- | --- | --- | --- |
| Set 01 | 0.88 (7/8) | 0.88 (14/16) | 1.00 (3/3) | 0.50 (1/2) | 0.90 (9/10) |
| Set 02 | 0.91 (10/11) | 0.79 (11/14) | 1.00 (2/2) | 1.00 (5/5) | 0.86 (6/7) |
| Set 03 | 0.93 (13/14) | 0.73 (8/11) | 0.50 (1/2) | 1.00 (6/6) | 0.67 (6/9) |
| Set 04 | 0.80 (8/10) | 0.64 (9/14) | 0.67 (2/3) | 0.60 (3/5) | 0.80 (8/10) |
| Set 05 | 0.67 (6/9) | 1.00 (16/16) | 1.00 (1/1) | 1.00 (1/1) | 1.00 (10/10) |
| Set 06 | 1.00 (10/10) | 0.79 (11/14) | 1.00 (4/4) | 1.00 (5/5) | 0.91 (10/11) |
| Set 07 | 1.00 (11/11) | 1.00 (13/13) | 1.00 (1/1) | 1.00 (6/6) | 1.00 (6/6) |
| Set 08 | 0.92 (12/13) | 0.80 (8/10) | 0.50 (1/2) | 1.00 (6/6) | 0.67 (6/9) |
| Set 09 | 0.93 (13/14) | 0.82 (9/11) | 1.00 (1/1) | 1.00 (5/5) | 0.89 (8/9) |
| Set 10 | 1.00 (13/13) | 0.73 (8/11) | 0.86 (6/7) | 1.00 (5/5) | 1.00 (11/11) |
| Total | 0.91 | 0.82 | 0.85 | 0.94 | 0.87 |

The first figure in the parentheses shows the correct detection by neural network, and the second figure shows the total number of patients with the corresponding infarction site. Acc = accuracy, Ant = anterior, Inf = inferior, MI = myocardial infarction, Post = posterior
